# The Mediating Effects of Social Support on the Relationship between Uncertainty and Quality of Life among Patients with Chronic Low Back Pain: A Cross-Sectional Survey

**DOI:** 10.3390/healthcare10091805

**Published:** 2022-09-19

**Authors:** Jin-Won Choi, Wi-Young So, Kyoung-Mi Kim

**Affiliations:** 1 Department of surgery, Pusan National University Hospital, Busan 49241, Korea; 2 Sports Medicine Major, College of Humanities and Arts, Korea National University of Transportation, Chungju-si 27469, Korea; 3 College of Nursing, Kosin University, Busan 49267, Korea

**Keywords:** chronic pain, mutual support system, prevalent symptom, psychological well-being, surgery

## Abstract

Background: This study aimed to investigate the mediating effects of social support on the relationship between uncertainty and quality of life (QOL) in patients with chronic low back pain (LBP). Methods: From 1 July 2019 to 25 March 2020, data were collected using a structured questionnaire from inpatients and outpatients > 20 years of age with chronic LBP lasting > 3 months. Inpatients included patients waiting for surgery and those recovering after surgery. The exclusion criteria were cancer and other serious pathological diseases. The relationships between uncertainty, social support, and QOL were analyzed using Pearson’s correlation coefficients. Results: Uncertainty, the independent variable, exerted a significant effect on social support, the mediator (B = 0.33, *p* < 0.001). In addition, both uncertainty (B = 0.37, *p* < 0.001) and social support (B = 0.45, *p* < 0.001) exerted statistically significant effects on QOL, the dependent variable. Conclusions: Disease-related uncertainty can reduce QOL in patients with chronic LBP, and this relationship is mediated by the level of social support. To develop strategies for strengthening social support from healthcare providers, family, and friends, future studies should examine the experiences of patients with chronic LBP from various perspectives, including pain intensity and duration.

## 1. Introduction

Low back pain (LBP), a highly prevalent symptom across all age groups worldwide [1], is both recurrent and persistent, frequently progressing to a chronic condition [2]. In South Korea, LBP represents the fifth most common cause of hospital visits, and >80% of individuals are reported to experience at least one episode of LBP in their lifetime [3]. LBP is a major contributor to premature mortality and disease burden due to disability [4]; according to a study that analyzed data from the 5^th^ Korea National Health and Nutrition Examination Survey (KNHANES), LBP is the most common chronic pain condition among both adult men and women [5].

Chronic LBP is not an illness but a symptom. Pain itself places a burden on the economically active population and on older adults, as it limits their range of activities [2]. Such restrictions in activity can lead to social isolation, which may be accompanied by psychological symptoms such as anxiety and depression [6,7]. Patients with chronic LBP tend to view pain negatively, and the passive management of pain may be associated with psychological complications such as depression, thereby leading to reductions in quality of life (QOL) [8]. Furthermore, patients with chronic LBP exhibit significantly poorer QOL than patients with acute LBP [7], highlighting the need for aggressive interventions targeting these measures.

Uncertainty is a key factor contributing to the deterioration of QOL in patients with chronic conditions [9]. In this context, uncertainty refers to a cognitive state provoked by uncertain therapeutic effects, unpredictable symptoms of disease, unclear explanations, unfamiliar events related to disease, and a lack of information related to disease progression—all of which are known to affect recovery [10]. Patients who undergo spinal surgery experience increased uncertainty due to preoperative symptoms such as LBP, sciatica, and intermittent claudication, as well as psychological challenges in adjusting to their normal everyday lives postoperatively if these symptoms persist. Research has indicated that levels of uncertainty among these patients parallel those observed in patients with heart conditions or cancer [11]. Symptom-related uncertainty exerts a substantial impact on functioning in patients with chronic LBP, especially in terms of employment [12]. Furthermore, uncertainty surrounding the therapeutic effects of spinal surgery, the persistence of preoperative symptoms after surgery, and concerns surrounding future triggers create negative emotions, which may hinder effective coping methods and adjustment in patients with chronic LBP [11].

Social support refers to the presence of a mutual support system regarding one’s health and encompasses all forms of positive resources obtained from others, including family, friends, and healthcare providers [13]. Patients with chronic pain develop resilience via positive social support, which may be associated with benefits during the disease process [14]. In contrast, negatively perceived social support may increase symptoms of emotional distress such as anxiety and depression [15], contributing to reductions in QOL [8]. These findings highlight the essential role of social support in reducing pain and discomfort. However, further studies are required to determine whether social support can aid in promoting optimal QOL in patients with chronic LBP.

Strong social support has attracted much attention as a crucial protective factor that promotes psychological well-being by reducing anxiety and depression in patients with chronic LBP [15]. Previous studies have reported that increasing perceptions of social support are associated with decreased disease-related uncertainty in patients with chronic illnesses [16,17]. In addition, perceived uncertainty exerts an adverse impact on health-related QOL [18].

Several studies have investigated QOL in patients with chronic conditions, highlighting uncertainty [11,18,19] and social support [18,19] as significant predictors of QOL. However, few have focused on patients with chronic LBP [7,20]. To address this issue and provide evidence that can be used to promote effective interventions, we aimed to investigate the mediating effects of social support on the relationship between uncertainty and QOL in patients with chronic LBP. Specifically, our study aimed to examine the following hypotheses:

**Hypothesis** **1:** *Uncertainty in patients with chronic LBP will have a significant effect on social support.*

**Hypothesis** **2:** *Uncertainty in patients with chronic LBP will have a significant effect on QOL.*

**Hypothesis** **3:** *Social support for patients with chronic LBP will have a significant effect on QOL.*

**Hypothesis** **4:** *Patients with chronic LBP will experience a mediating effect from social support on the relationship between uncertainty and QOL.*

## 2. Materials and Methods

### 2.1. Research Groups

The current study was conducted among neurosurgery inpatients and outpatients with chronic LBP lasting ≥ 3 months treated at Pusan National University Hospital, a large hospital in Busan City with a capacity of 1000 beds. Inpatients included patients waiting for surgery and patients recovering after surgery. The patients were aged > 20 years and had non-serious back pain. Patients with cancer and other serious pathological diseases were excluded. Inpatients and outpatients of the hospital were those whose pain lasted > 3 months and for whom the pain was persistent and moderate to severe. Patients were diagnosed with LBP by a hospital doctor. The inclusion and exclusion criteria were as follows:

The inclusion criteria

- Neurosurgery inpatients and outpatients with chronic LBP lasting ≥ 3 months;
- Patients waiting for surgery and patients recovering after surgery;
- Non-serious back pain;
- Aged > 20 years;
- Pain was persistent and moderate to severe.

The exclusion criteria

- Cancer and other serious pathological diseases;
- Aged < 20 years.

### 2.2. QOL

QOL was measured using the World Health Organization‘s QOL Instruments—Short Version (WHOQOL-BREF) [21] translated by Min et al. [22]. This tool consists of 26 items across five domains: physical health, psychological health, social health, environmental health, and general health. Each item is rated on a scale from 1 (“not at all”) to 5 (“completely”), with higher total scores indicating higher QOL. The reliability of the tool (Cronbach’s α) was 0.89 at the time of development [21] and 0.81 in this study. The small sample size was considered to have reduced the reliability value.

### 2.3. Uncertainty

Uncertainty was measured using the Mishel Uncertainty in Illness Scale (MUIS) [10], which was modified and translated by Chung et al. [23]. This 33-item tool comprises four factors: unpredictability of disease and prognosis, ambiguity of disease, inconsistency of diagnosis and disease severity, and complexity of the care system and treatment. Each item is rated on a scale from 1 (“strongly disagree”) to 5 (“strongly agree”), with higher total scores indicating a higher level of uncertainty. The reliability of the tool (Cronbach’s α) was 0.91–0.93 at the time of development by Mishel [10] and 0.70 in this study.

### 2.4. Social Support

Social support was measured using the Multidimensional Scale of Perceived Social Support (MSPSS) developed by Zimet et al. [24], which was modified and translated by Shin and Lee [25]. This 12-item tool assesses social support across three areas: family, friends, and special support (including healthcare providers [26]). Each item is rated on a scale from 1 (“strongly disagree”) to 5 (“strongly agree”), with higher total scores indicating a higher degree of social support. The reliability of the tool (Cronbach’s α) was 0.85 at the time of development [24] and 0.84 in this study.

### 2.5. Data Collection Process

Data were collected from 1 June 2019 to 25 March 2020. This study was approved by the institutional review board of Kosin University, Busan, Korea (No: IRB 2019-0040). Before collecting data, we contacted the nursing department and relevant medical department at Pusan University in Busan Metropolitan City to explain the purpose, method, and procedures of the study and to obtain permission and cooperation. The purpose of this study and the content of the experiment were explained to the participants, who subsequently provided written informed consent prior to study commencement.

### 2.6. Statistical Analysis

The sample size for the regression analysis was determined using G-Power software (G-power program 3.1.9.7, Heinrich-Heine-University, Düsseldorf, Germany). Using a significance level of 0.05, power of 95%, moderate effect size of 0.15, and seven predictors, the minimum sample size was calculated as 153. Considering 10% potential withdrawals, the questionnaire was administered to 165 participants. After excluding 10 participants with cancer (n = 3), serious pathological diseases (n = 5), and ages < 20 years (n = 2), 155 were included in the final analysis. The survey was conducted in wards and outpatient clinics using a paper-based questionnaire.

The collected data were analyzed using IBM SPSS/WIN for Windows, version 25.0, and SPSS PROCESS macro 3.5 version software (IBM Corp., Armonk, NY, USA). General characteristics were analyzed as frequencies and percentages and as the mean and standard deviation for major variables. The differences in uncertainty, social support, and QOL according to general characteristics were analyzed using *t*-tests, one-way analyses of variance (ANOVA), and Scheffe’s test for post hoc comparison. The relationships between uncertainty, social support, and QOL were analyzed using Pearson’s correlation coefficients. The significance of the indirect effect was verified via bootstrap analysis in PROCESS macro using 10,000 bootstrap samples and a bias-corrected 95% confidence interval. Statistical significance was set at α = 0.05.

## 3. Results

### 3.1. General Characteristics

**Figure 1:**
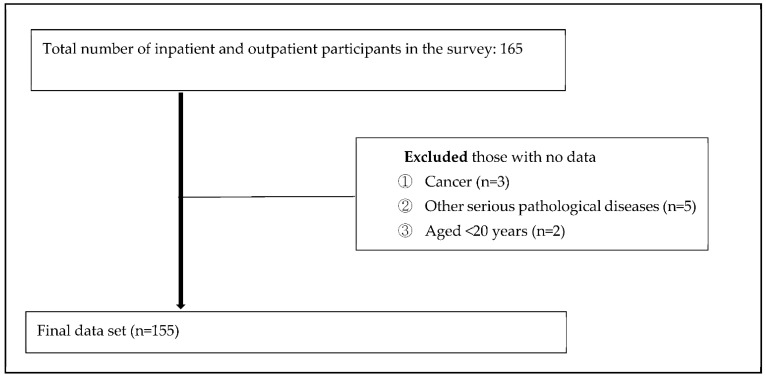
Flowchart of patients with chronic low back pain in this study.

A total of 155 participants were enrolled (Figure 1), including 89 men (57.4%) and 66 women (42.6%). Overall, 22 participants were under the age of 40 years (14.2%), 38 in their 40s (24.5%), 33 in their 50s (21.3%), 43 in their 60s (27.7%), and 19 in their 70s or older (12.3%). A total of 117 (75.5%) patients were employed, while 38 (24.5%) were not. The weekly frequency of LBP episodes was as follows: 1–2 times (n = 19, 12.3%), 3–4 times (n = 38, 24.5%), 5–6 times (n = 43, 27.7%), and ≥7 times (n = 55, 35.5%). Among the included patients, 116 had a pre-existing condition (74.8%), while 39 did not (25.2%) (Table 1).

### 3.2. Differences in Uncertainty, Social Support, and QOL according to General Characteristics

There were significant differences in uncertainty according to employment status and LBP frequency. The post hoc test confirmed that uncertainty levels were significantly higher in participants with a weekly LBP frequency of 1–2 times or 3–4 times than in those with a weekly LBP frequency of 5–6 times or ≥7 times. There were significant differences in social support according to pre-existing conditions. In addition, there were significant differences in QOL according to employment status, LBP frequency, and the presence of pre-existing conditions. The post hoc test confirmed that QOL ratings were significantly higher in participants with a weekly LBP frequency of 3–4 times than in those with a frequency of ≥7 times (Table 1).

### 3.3. Correlations among Uncertainty, Social Support, and QOL

Negative correlations were observed between uncertainty and QOL (r = −0.32, *p* < 0.001) and between uncertainty and social support (r = −0.41, *p* < 0.001). There was a positive correlation between social support and QOL (r = 0.45, *p* < 0.001) (Table 2).

### 3.4. Mediating Effect of Social Support on the Relationship between Uncertainty and QOL

Before analyzing the mediating effect of social support on the relationship between uncertainty and QOL, we evaluated multicollinearity among the independent variables. The tolerance values ranged from 0.834–0.857, and the variance inflation factor (VIF) was <1.17, thereby satisfying the criterion of ≤10 [27]. The correlations between the independent variables ranged from 0.32–0.45, all of which were <0.80, thereby confirming the absence of multicollinearity. The Durbin–Watson statistic was 1.82, which was close to the cutoff of 2.00 [27], confirming the absence of autocorrelation in the dependent variable. In the descriptive analysis, employment status, frequency of LBP, and comorbidity exhibited a significant relationship with QOL. Thus, these variables were included as control factors in the mediation analysis. Uncertainty, the independent variable, exerted a significant effect on social support, the mediator (B = 0.33, *p* < 0.001). In addition, both uncertainty (B = 0.37, *p* < 0.001) and social support (B = 0.45, *p* < 0.001) exerted statistically significant effects on QOL, the dependent variable (Table 3).

**Figure 2:**
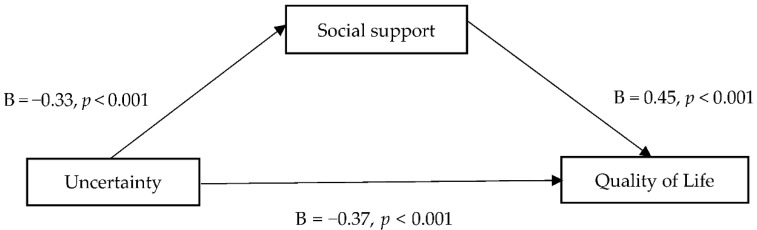
Mediating effects of social support on the relationship between uncertainty and quality of life.

Table 4 shows the direct and indirect effects of uncertainty on QOL. First, the size of the direct effect of uncertainty on QOL was −0.37 (*p* < 0.001), and the 95% bootstrap CI (0.33–0.42) did not include 0, confirming statistical significance. The size of the indirect effect of uncertainty on QOL through social support was 0.15 (*p* < 0.001), and the 95% bootstrap CI (0.08–0.21) did not include 0, confirming statistical significance. Figure 2 illustrates the relationships between uncertainty, social support, and QOL. A concise and precise description of the experimental results, their interpretation, and the experimental conclusions are provided.

Last, the multivariate linear regression model examines predictors of QOL. The overall model was statistically significant: *F* = 10.768, *p* < 0.001. Furthermore, the model explained >30% (*R*^2^ = 0.34, Adjusted *R*^2^ = 0.31) of the variance in the dependent variable of QOL (Table 5).

## 4. Discussion

In this study, we investigated the mediating effects of social support on the relationship between uncertainty and QOL in patients with chronic LBP. The major finding of the study was that uncertainty exerts a direct influence on QOL in patients with chronic LBP, as well as an indirect influence via the mediation of social support. Our results showed that the level of uncertainty was higher among patients with a weekly LBP frequency of 1–2 or 3–4 times than among those with a frequency of 5–6 times or ≥7 times, suggesting that uncertainty levels are higher among those with less frequent LBP.

QOL significantly differed according to employment status and the presence of a pre-existing condition. This result is consistent with previous reports of relatively higher levels of emotional distress (e.g., depression, anxiety) among patients with LBP who are unemployed or have pre-existing conditions [15]. Furthermore, our findings are in accordance with the results of Lee and Kim [18], who reported higher QOL among employed patients with peripheral artery disease than among their unemployed counterparts. However, in contrast to our results, Lee and Kim [18] reported no differences in QOL according to the presence of a pre-existing condition. This may be because individual cardiovascular diseases such as high blood pressure exert significant effects on QOL in patients with peripheral artery disease, which may confound this association. Our results suggested that the presence of underlying diseases negatively impacts QOL in patients with chronic LBP, highlighting the need to devise strategies for boosting QOL in these patients. Our results also indicated that the patients with a pain frequency ≥ 7 times per week had significantly poorer QOL than those with a pain frequency of 3–4 times a week. Accordingly, previous reports have demonstrated that pain intensity increases with increasing LBP frequency and duration [1] and that the duration of pain is negatively correlated with QOL in patients with chronic LBP [7]. Subsequent studies should therefore consider the duration of pain during their analysis.

Our results also demonstrated a significant effect of uncertainty on social support. Specifically, perceived social support decreased with increasing uncertainty. This finding is consistent with the results of a previous study that reported a correlation between social support and uncertainty among patients undergoing hemodialysis [17], women who were cancer survivors [28] and pregnant [29], and patients with cholangiocarcinoma [30] and stroke [31]. However, our results contrast with those reported in another study that reported no significant association between uncertainty and social support among patients with peripheral artery disease [18], elderly patients with cancer [19], and those with Parkinson’s disease [32]. This inconsistency may be related to the mean ages of participants in the different study samples. The mean ages in both our study and the previous study [17] were 54 and 53 years, respectively, while those in other studies [18,19] were ≥65 years and 69 years, respectively. Our study participants were mostly middle-aged adults who received social support from various sources; this support tends to be lower among older adults [33]. Further studies should address this issue and examine the relationship between social support and uncertainty in the context of age. In addition, in future studies, it is necessary to compare the relationship between uncertainty and social support according to disease.

In our study, QOL significantly decreased with increasing uncertainty. This result is in line with the findings of previous studies [19,33] that reported a negative correlation between uncertainty and QOL. Our findings are also in accordance with those of Lee and Kim [18], who identified uncertainty as a significant predictor of QOL among patients with peripheral artery disease. Other studies have reported that patients who undergo spinal surgery experience increased uncertainty as their level of knowledge related to self-care decreases [11]. Many patients who seek medical care for LBP wish to undergo a procedure without taking the prescribed analgesic, based on the belief that drugs are bad for the body [1]; systematic education is necessary to increase the level of knowledge and reduce uncertainty in patients with chronic LBP. Such reductions in uncertainty may help to improve QOL in these patients.

Our results also demonstrated that social support mediates the relationship between uncertainty and QOL. Previous studies have reported associations between uncertainty and social support [17] and that uncertainty predicts QOL [18]. Our findings extend those of previous studies [17,18], highlighting the key role of social support in the relationship between uncertainty and QOL among patients with chronic LBP. However, previous studies were conducted among patients with various chronic diseases, making direct comparisons of the findings difficult. Nonetheless, our findings are similar to those reported by Kim and Choi [16], who demonstrated that providing appropriate social support can reduce uncertainty among patients undergoing hemodialysis. Thus, treatment planning and counseling for patients with chronic LBP should focus on interventions that can enhance social support to reduce uncertainty and improve QOL.

Our study had some limitations, including its small sample size, which requires validation of the findings in larger populations. Notably, the population comprised patients with chronic LBP, and weekly LBP frequency was examined with reference to a previous study [6]. Although we differentiated our patients from those experiencing chronic LBP every day, we did not consider the influence of pain intensity. Therefore, subsequent studies should address this issue to examine pain from multiple perspectives. In addition, a study by Du et al. [34] targeting patients with chronic non-specific LBP reported that social support was negatively correlated with emotional distress, highlighting the need for additional studies to examine this relationship. Finally, since our study was conducted in only one city in Korea, it cannot be generalized to represent the entire population of Korea, other countries, or cultures.

## 5. Conclusions

Disease-related uncertainty can reduce QOL in patients with chronic LBP, and this relationship is mediated by the level of social support. To develop strategies for strengthening social support from healthcare providers, family, and friends, future studies should examine the experiences of patients with chronic LBP from various perspectives, including pain intensity and duration. Additional studies should investigate the effectiveness of multidisciplinary interventions that reduce uncertainty and strengthen social support in these patients.

## Figures and Tables

**Table 1:** Uncertainty, social support, and quality of life according to the general characteristics of participants (n = 155).

Characteristics	Categories	n (%)	Uncertainty	*t* or F	*p* (Post hoc)	Social Support	*t* or F	*p* (Post hoc)	Quality of Life	*t* or F	*p* (Post hoc)
Sex	Men	89 (57.4)	2.49 ± 0.51	−0.77	0.441	3.24 ± 0.46	1.73	0.086	2.96 ± 0.34	0.54	0.592
	Women	66 (42.6)	2.55 ± 0.54			3.11 ± 0.45			2.93 ± 0.38		
Age (years)	<40	22 (14.2)	2.69 ± 0.66	0.72	0.581	3.29 ± 0.53	0.67	0.614	3.10 ± 0.42	1.64	0.168
	40~49	38 (24.5)	2.52 ± 0.49			3.16 ± 0.37			2.98 ± 0.32		
	50~59	33 (21.3)	2.46 ± 0.37			3.13 ± 0.43			2.88 ± 0.28		
	60–69	43 (27.7)	2.49 ± 0.60			3.11 ± 0.44			2.89 ± 0.38		
	≥70	19 (12.3)	2.49 ± 0.49			3.22 ± 0.60			2.93 ± 0.42		
Employment status	Yes	117 (75.5)	2.60 ± 0.51	−3.25	0.002 **	3.21 ± 0.46	−1.74	0.084	2.98 ± 0.37	−2.19	0.030 *
	No	38 (24.5)	2.29 ± 0.53			3.05 ± 0.43			2.83 ± 0.31		
Frequency of low back pain (times/week)	1–2 (a)	19 (12.3)	2.86 ± 0.42	9.31	<0.001 *** (c, d < a, b)	3.20 ± 0.42	2.23	0.087	3.03 ± 0.28	5.28	0.002 ** (d < b)
	3–4 (b)	38 (24.5)	2.76 ± 0.46			3.27 ± 0.45			3.08 ± 0.34		
	5–6 (c)	43 (27.7)	2.43 ± 0.50			3.22 ± 0.38			2.96 ± 0.35		
	≥7 (d)	55 (35.5)	2.33 ± 0.52			3.05 ± 0.51			2.81 ± 0.38		
Comorbidity	Yes	116 (74.8)	2.51 ± 0.51	−0.58	0.566	3.12 ± 0.46	−2.07	0.040 *	2.91 ± 0.36	−2.04	0.043 *
	No	39 (25.2)	2.57 ± 0.59			3.29 ± 0.44			3.05 ± 0.36		

Data are expressed as mean ± standard deviation; * *p* < 0.05, ** *p* < 0.01, *** *p* < 0.001; tested by independent *t*-test or one-way analyses of variance (Scheffe post hoc test).

**Table 2:** Correlations between uncertainty, social support, and quality of life (n = 155).

Variables	Uncertainty	Social Support
	r (*p)*	r (*p)*
Uncertainty	1.00	
Social support	−0.41 (<0.001 ***)	1.00
Quality of life	−0.32 (<0.001 ***)	0.45 (<0.001 ***)

*** *p* < 0.001; tested by Pearson’s correlation coefficients.

**Table 3:** Mediating effect of social support on the relationship between uncertainty and quality of life (n = 155).

Models	Variables			B	SE	*t*	*p*	95% CI
Model 1	Uncertainty	→	Social support	−0.33	0.06	−5.05	<0.001 ***	0.20–0.46
Model 2	Uncertainty	→	Quality of life	−0.37	0.02	−16.65	<0.001 ***	0.33–0.42
Model 3	Social support	→	Quality of life	0.45	0.03	17.34	<0.001 ***	0.40–0.50

B = regression weights; SE = standardized error; CI = confidence interval. *** *p* < 0.001; tested by bootstrap analysis.

**Table 4:** Direct and indirect effects on quality of life (n = 155).

Variables	Direct Effect					Indirect Effect				
	B	Boot SE	95% CI			B	Boot SE	95% CI		
			Boot LLCI	Boot ULCI	*p*			Boot LLCI	Boot ULCI	*p*
Uncertainty → Quality of life	−0.37	0.02	0.33	0.42	<0.001 ***					
Uncertainty → Social support → Quality of life						0.15	0.03	0.08	0.21	<0.001 ***

CI = confidence interval; SE = standardized error; LLCI = the lower limit of B in 95% confidence interval; ULCI = the upper limit of B in 95% confidence interval tested by bootstrap analysis; *** *p* < 0.001; tested by bootstrap analysis.

**Table 5:** Multiple linear regression of quality of life (n = 155).

Variables	Unstandardized Beta	Standard Error	Standardized Beta	t	*p*
Uncertainty	−0.14	0.08	−0.14	−1.81	0.072
Social support	0.54	0.10	0.39	5.31	0.000
Sex	−1.34	1.29	−0.07	−1.04	0.301
Age	−0.05	0.05	−0.07	−0.96	0.338
Employment status	−2.34	1.49	−0.11	−1.57	0.118
Frequency of low back pain	−2.01	0.62	−0.22	−3.25	0.001
Comorbidity (No)	3.41	1.58	0.16	2.16	0.033

Model: *R*^2^ = 0.34; adjusted *R*^2^ = 0.31; F = 10.768; *p* < 0.001.

## Data Availability

The data presented in this study are available upon request from the authors. Some variables were restricted to preserve the anonymity of study participants.

## References

[1] Kim S.K., Kim H.S., Chung S.S. (2017). Degrees of Low Back Pain, Knowledge of and Educational Needs for Low Back Pain in Patients with Chronic Low Back Pain. Korean Soc. Muscle Jt. Health.

[2] Hartvigsen J., Hancock M.J., Kongsted A., Louw Q., Ferreira M.L., Genevay S., Woolf A. (2018). What low back pain is and why we need to pay attention. Lancet.

[3] Juch J.N., Maas E.T., Ostelo R.W., Groeneweg J.G., Kallewaard J.W., Koes B.W., Van Tulder M.W. (2017). Effect of radiofrequency denervation on pain intensity among patients with chronic low back pain: The mint randomized clinical trials. Jama.

[4] Korea Disease Control and Prevention Agency (2020). 2020 Status and Issues of Chronic Diseases. Policy Research.

[5] Jeong E.K., Kwak Y.H., Song J.S. (2015). Influences of Chronic Pain on the Use of Medical Services in South Korea. J. Korea Contents Assoc..

[6] Ahn J.H., Kim H.S., Kim H.J. (2019). Pain, Disability, Emotional Status and Educational Needs between Acute and Chronic Low Back Pain Groups. J. Korean Biol. Nurs. Sci..

[7] Hong J.H., Kim H.D., Shin H.H., Huh B. (2014). Assessment of depression, anxiety, sleep disturbance, and quality of life in patients with chronic low back pain in Korea. Korean J. Anesthesiol..

[8] Kim K.A., Chu S.H. (2017). Mediating Effect of Coping Strategies in the Relationship between Pain Beliefs and Depression, Pain Disability among Chronic Back Pain Patients. J. Korea Contents Assoc..

[9] Cha K.S., Kim K.H. (2012). Impact of Uncertainty on Resilience in Cancer Patients. Korean Oncol. Nurs. Soc..

[10] Mishel M.H. (1988). Uncertainty in illness. J. Nurs. Scholarsh..

[11] Jun M.H., Jung J.Y., Kim M.S. (2012). Factors Affecting Post-operative Uncertainty of the Patients Undergone Lumbar Spinal Surgery. Korean Soc. Muscle Jt. Health.

[12] Bunzli S., Watkins R., Smith A., Schütze R., O’Sullivan P. (2013). Lives on hold: A qualitative synthesis exploring the experience of chronic low-back pain. Clin. J. Pain.

[13] Ferrans C.E., Zerwic J.J., Wilbur J.E., Larson J.L. (2005). Conceptual model of health-related quality of life. J. Nurs. Scholarsh..

[14] Sturgeon J.A., Zautra A.J. (2010). Resilience: A new paradigm for adaptation to chronic pain. Curr. Pain Headache Rep..

[15] Du S., Hu Y., Bai Y., Hu L., Dong J., Jin S., Zhang H. (2019). Emotional distress correlates among patients with chronic nonspecific low back pain: A hierarchical linear regression analysis. Pain Pract..

[16] Kim Y.J., Choi H.J. (2012). The Influence of Uncertainty and Social Support on General Well-being among Hemodialysis Patients. Korean J. Rehabil. Nurs..

[17] Kim B.M., Kim J.H. (2019). Influence Of Uncertainty, Depression, And Social Support On Self-Care Compliance In Hemodialysis Patients. Ther. Clin. Risk Manag..

[18] Lee H.J., Kim Y.K. (2020). Effects of Uncertainty, Social Support, and Sick Role Behavior on Health-Related Quality of Life in Patients with Peripheral Arterial Disease. J. Korean Clin. Nurs. Res..

[19] Kim K.O., Kim J.A. (2017). Influences of Uncertainty and Social Support on the Quality of Life among Elderly Cancer Patients. Korean Oncol. Nurs. Soc..

[20] Soer R., Reneman M.F., Speijer B.L., Coppes M.H., Vroomen P.C. (2012). Clinimetric properties of the EuroQol-5D in patients with chronic low back pain. Spine J..

[21] Whoqol Group (1998). Development of the World Health Organization WHOQOL-BREF quality of life assessment. Psychol. Med..

[22] Min S.K., Lee C.I., Kim K.I., Suh S.Y., Kim D.K. (2000). Development of Korean Version of WHO Quality of Life Scale Abbreviated Version(WHOQOL-BREF). J. Korean Neuropsychiatr. Assoc..

[23] Chung C.W., Kim M.J., Rhee M.H., Do H.G. (2005). Functional Status and Psychosocial Adjustmentin Gynecologic Cancer Patients Receiving Chemotherapy. Korean J. Women Health Nurs..

[24] Zimet G.D., Dahlem N.W., Zimet S.G., Farley G.K. (1988). The multidimensional scale of perceived social support. J. Personal. Assess..

[25] Shin J.S., Lee Y.B. (1999). The Effect of Social Supports on Psychosocial Well-being of the Unemployed. Korean J. Soc. Welf..

[26] Kim Y.J., Lee K.J. (2010). Relationship of Social Support and Meaning of Life to Suicidal Thoughts in Cancer Patients. J. Korean Acad. Nurs..

[27] Eyduran E., Ozdemir T., Alarslan E. (2005). Importance of diagnostics in multiple regression analysis. J. Appl. Sci..

[28] Lee I.S., Park C.S. (2020). The mediating effect of social support on uncertainty in illness and quality of life of female cancer survivors: A cross-sectional study. Health Qual. Life Outcomes.

[29] Hui Choi W.H., Lee G.L., Chan C.H., Cheung R.Y., Lee I.L., Chan C.L. (2012). The relationships of social support, uncertainty, self-efficacy, and commitment to prenatal psychosocial adaptation. J. Adv. Nurs..

[30] Somjaivong B., Thanasilp S., Preechawong S., Sloan R. (2011). The influence of symptoms, social support, uncertainty, and coping on health-related quality of life among cholangiocarcinoma patients in northeast Thailand. Cancer Nurs..

[31] Zhang H., Gao J., Sun Y., Han Z. (2020). Coping Styles and Stroke Knowledge Mediate the Effect of Social Support and Uncertainty Among Stroke Patients. Innov. Aging.

[32] Chung S., Kim E.J., Houston J.B. (2021). Perceived online social support for Parkinson’s disease patients: The role of support type, uncertainty, contentment, and psychological quality of life. Commun. Q..

[33] Chen Y., Feeley T.H. (2014). Social support, social strain, loneliness, and well-being among older adults: An analysis of the Health and Retirement Study. J. Soc. Pers. Relatsh..

[34] Du S., Hu L., Dong J., Xu G., Chen X., Jin S., Zhang H., Yin H. (2017). Self-management program for chronic low back pain: A systematic review and meta-analysis. Patient Educ. Couns..

